# Dissemination and implementation analysis of the Ross procedure in adults: time to update the guidelines?

**DOI:** 10.1186/s43057-023-00119-5

**Published:** 2023-12-20

**Authors:** Kyle S. Bilodeau, David C. Mauchley, Scott DeRoo, Christopher R. Burke

**Affiliations:** 1 Department of Surgery, Division of Cardiothoracic Surgery, University of Washington, Seattle, WA USA; 2 Department of Surgery, Division of Cardiac Surgery, Seattle Children’s Hospital, Seattle, WA USA

**Keywords:** Valvular heart disease, Aortic valve disease, Ross procedure, Implementation science

## Abstract

**Background:**

The science of dissemination and implementation (D&I) aims to improve the quality and effectiveness of care by addressing the challenges of incorporating research and evidence-based practice into routine clinical practice. This lens of D&I has challenged the interpretation and incorporation of data, noting that failure of a given therapy may not reflect lack of efficacy, but instead reflect an imperfect implementation. The aim of this manuscript is to review the influence of the Ross procedure’s historical context on its D&I.

**Methods:**

A contextual baseline of the Ross procedure was defined from the procedure’s original description in the literature to major publications since the 2017 valvular heart disease guidelines. D&I evaluation was conducted using the Consolidated Framework for Implementation Research (CFIR), using constructs from each of the five respective domains to define the main determinants.

**Results:**

Each of the five CFIR domains appears to be correlated with a factor influencing the Ross procedure’s varied history of enthusiasm and acceptance. The complex nature of Ross required adaptation for optimization, with a strong correlation of center volume on outcomes that were not considered in non-contemporary studies. Outcomes later published from those studies influenced social and cultural contexts within the aortic surgery community, and led to further organizational uncertainty, resulting in slow guideline incorporation.

**Conclusions:**

The D&I of the Ross procedure was a result of inadequate appreciation of technical complexity, effect of patient selection, and complex aortic surgery experience, resulting in dismissal of an efficacious procedure due to a misunderstanding of effectiveness.

## Background

The science of dissemination and implementation (D&I) has emerged as a novel field, with an aim to improve the quality and effectiveness of care by addressing the challenges of incorporating research and evidence-based practice into routine clinical practice [1]. This lens of D&I has challenged the interpretation and incorporation of data, as it has shifted the focus toward gauging effectiveness over efficacy [2]. An example of this shift was eloquently stated by Heiden et al., noting that while traditional research has focused on efficacy, such as the characterization of a drug or intervention within a controlled study environment, effectiveness is how that drug or intervention benefits a given population of individuals within the real-world [3]. They note failure of a given therapy may not reflect lack of efficacy, but instead reflect an imperfect implementation of said therapy within a given population [3]. Therefore, focusing on the context and outcomes of implementation provides substantial insight into a therapy’s observed clinical outcomes [3].

The Ross procedure is a technically complex operation to address pathology of the aortic valve, whereby the patient’s own pulmonary valve (autograft) is used to replace the diseased aortic valve. Although originally described by Donald Ross in the 1960s, this operation has yielded a complex history and varied acceptance within the cardiovascular community. A notable theme arises when analyzing the historical context and implementation outcomes of the Ross procedure, noting initial enthusiasm was later met with skepticism after publications of poor outcomes and complications led to concerns of feasibility, which in turn created hesitation to adopt Ross as a treatment option for adult aortic valvular heart disease [4, 5]. A recent systematic review and meta-analysis sought to compare the Ross procedure with mechanical aortic valve replacement (mAVR) in adult patients, citing that the Ross procedure was found to have improved freedom from all-cause mortality and challenging decades of concerns over the perception that the Ross procedure imposes increased surgical risk and high rates of reintervention [6–8]. Thus, the dichotomy between recent data and existing guidelines again frames the question of what is the optimal aortic valve substitute in young and middle-aged adults requiring aortic valve surgery? The aim of this manuscript is to review the historical context of the Ross procedure, highlighting the past and current evidence, as well as to characterize the barriers to dissemination and implementation of Ross within current practice.

## Methods

Evaluation of the Ross procedure was conducted through application of the Consolidated Framework for Implementation Research (CFIR) originally published by Damscroder et al. [9]. CFIR was created to provide a pragmatic structure to guide formative evaluations in the real world, highlighting that utilization of outcomes alone are inadequate, as researchers and practitioners must recognize that the success of an intervention is also dependent on its implementation and optimization within a given system. CFIR accomplishes this by outlining five major domains, all of which interact and influence the overall effectiveness of implementation, noting that implementation is inherently a social process and includes a provider’s perception of the evidence (Table 1) [9]. This evaluation was performed in two phases: defining of a contextual baseline and evaluation of the D&I of the Ross procedure using applied domains from CFIR. The contextual baseline was summative in nature, focusing on a brief review of the Ross procedure from its original publication, evidence influencing current valvular heart disease guidelines, and major publications since the 2017 guideline release. Evaluation of the Ross procedure’s D&I was performed using constructs from each of the five respective CFIR domains, with emphasis placed on defining and analyzing D&I determinants.

**Table 1:** Overview of selected domains from the Consolidated Framework for Implementation (adapted from Damschroder et al. [9])

Intervention characteristics	• Complex • Multi-faceted • Require active engagement of individuals • Require adaptation
Outer setting	• Economic, political, and social • Context within which an organization resides
Inner setting	• Structural, political, and cultural • Context through which the implementation process will proceed
Individuals involved	• Individuals possess agency in power/influence • Includes personal, organizational, and professional mindsets, norms, and affiliations
Implementation process	• Active change process • Aimed to achieve individual and organization use of an intervention

## Results

### Historical context of the Ross procedure

Donald Ross first described the Ross procedure in 1967, where replacement of the diseased aortic valve was accomplished via a pulmonary autograft and subsequent placement of a pulmonary homograft [10]. The Ross procedure’s novelty was that it not only alleviated the requirement of life-long anticoagulation, but that it created a living valvular substitute with potential to confer an adaptive physiologic functionality and hemodynamic profile more akin to the native aortic valve [11]. Realization of such potential benefits led to enthusiasm in the 1990s; however, later reports of aortic insufficiency in the setting of neo-aortic root dilation led to waning interest in the surgical community [12–14]. The estimated peak of Ross procedures within North American was postulated to be circa 1998, when it accounted for approximately 1.2% of aortic valve replacements (AVR) and was then followed by a steady decline through 2010 [7]. During this era of decline, multiple studies conveyed concern regarding the efficacy of the Ross procedure and further contributed to the ongoing decline in implementation into clinical practice [8, 15, 16]. In 2014, Reece et al. performed a propensity-matched analysis from the STS database, publishing findings that the Ross procedure was associated with a 3-fold higher operative mortality comparted to conventional AVR (2.7% vs. 0.9%) [7]; however, Mazine and El-Hamamsy diligently note that the median annual number of Ross procedures performed per center was less than 1, and only 6 of the 231 centers analyzed had performed greater than or equal to 5 Ross procedures annually, raising concern for confounding given prior data on aortic root surgery volume and outcomes [17, 18]. Thus, much of the data from the early 2000s and 2010s portrayed the Ross procedure as an alternative with limited durability and a potentially excess peri-operative mortality amongst younger, non-elderly populations with aortic valve disease requiring AVR.

### The dichotomy between contemporary evidence and current guidelines for valvular heart disease

Both the American Heart Association/American College of Cardiology (AHA/ACC) and European Society of Cardiology (ESC) issued new guidelines on valvular heart disease in 2020 and 2021, respectively. The AHA/ACC guidelines recommend use of mechanical values in patients younger than 50 years, bioprosthetic valves in patients older than 65 years, and surgeon/patient choice for patients aged between 50 and 65 years, while the ESC guidelines recommend a mechanical valve in patients younger than 60 years and bioprosthetic in patients older than 65 years [19, 20]. Both AHA and ESC guidelines have been based on two randomized controlled trials from the 1970s and 1980s, as well as a more recent randomized trial and some observational studies [17, 21–23]. Despite having been published within 1 year, the subtle difference of recommendation by age reflects the ongoing uncertainty associated with valve replacement in non-elderly patients [17]. AHA/ACC has given the Ross procedure a class IIb recommendation in both the 2017 and 2020 guidelines, whereas the ESC now acknowledge Ross as an alternative therapy in select patients after failing to acknowledge Ross as a surgical option in their 2017 guidelines [19, 20]. To date, the Canadian Cardiovascular Society remains the only other society with a recommendation to consider Ross in non-elderly patients undergoing AVR [24].

Since publication of the 2017 AHA/ACC and ESC guidelines, a study by Goldstone et al. comparing biologic vs. mechanical prostheses reported considerable mortality amongst valve recipients aged 45–54 (30.6% vs. 26.4% over 17 years, respectively), challenging decades of prior research and concluding that the marginal mortality benefits in younger patients who receive mechanical valves are significantly outweighed by higher rates of bleeding and stroke [25]. They additionally note that evidence regarding prosthesis selection had previously assumed equal mortality; however, their results conclude that most prior studies were likely underpowered to detect any clinically relevant differences within populations [25]. In 2018, Mazine et al. published the most up-to-date systematic review and meta-analysis comparing Ross and mAVR [6]. They report that compared to patients who underwent mAVR, patients who received a Ross had a 46% reduction in all-cause mortality (*p* = 0.04) and a lower incidence of valve- or cardiac-related mortality (0.04% vs. 0.09%, *p* = 0.04). The rates of perioperative mortality were not statistically significant between groups (*p* =0.36); however, Ross was noted to have a lower incidence of postoperative heart block requiring permanent pacemaker implantation (*p* = 0.04), and lower rates of stroke (*p* = 0.02) and major bleeding (*p* < 0.001) at follow-up. Ross was additionally associated with improved quality of life and hemodynamics, as well as higher scores within the subdomains of bodily pain, social functioning, and mental health, which they attributed to a combination of alleviation of the need for chronic anticoagulation and a pulmonary autograft with innate biologic properties capable of performing native root functionality. Their findings overall were congruent with numerous contemporary studies all demonstrating favorable data in support of Ross [6, 17, 26–34].

### Context of Ross implementation and outcomes

When examining the Ross procedure utilizing CFIR domains and thus a lens of implementation, each of the five domains appears to be correlated with a factor influencing the Ross procedure’s varied history of enthusiasm and acceptance (Table 2). The technical conduct of the Ross procedure itself is undeniably complex. Successful performance of the operation requires a deep understanding of aortic root surgery and the technical components require extensive (sometimes hazardous) suture lines that respect the symmetry of the autograft in multiple dimensions. Many of the components of the operations are unique regarding the typical “skill set” of an adult cardiac surgeon (autograft harvest, autograft implantation, pulmonary artery-aortic anastomosis, reconstruction of the right ventricular outflow tract [RVOT]). The analysis of Society of Thoracic Surgeons outcomes data published by Reece et al. citing a 3-fold higher operative mortality was notable as the median Ross procedures performed per center was less than 1; poor outcomes observed in this series were likely heavily influenced by lower volume centers. A case volume-outcome relationship has been consistently shown in aortic root surgery, and certainly, the Ross procedure should be no exception [17, 18]. Inherent to this domain is a concept that without adaptation, interventions typically come to be seen as a poor fit and are thus resisted; however, with adaptation, complex and multi-faceted interventions can be modified to fit their intended setting/use [9]. This highlights the importance of proper program establishment (ensuring suitable support and volume and avoidance of very low volume centers), surgeon training (expertise and skills acquisition), and continuous self-audit processes to ensure excellence and integrity in outcomes. Initial experience and observations of late technical failures have been investigated and insights into technical modifications and selection criteria published by multiple authors, which has likely led to improved outcomes and thus supports that initial outcomes data alone are not sufficient for determining the efficacy of the Ross procedure [6, 17, 26, 33].

**Table 2:** Characterization of the Ross procedure using an implementation lens

Intervention characteristics	• Complex procedure • Required adaptation of surgical techniques for optimization • Influence of center volume on outcomes
Inner and outer setting	• Complications and poor outcomes imbued social and cultural concerns on safety/efficacy leading to abandonment and disillusionment
Individuals involved	• Organizational uncertainty • Influence on provider perceptions • Further confusion over procedural efficacy
Implementation process	• Slow incorporation into guideline

**Fig. 1:**
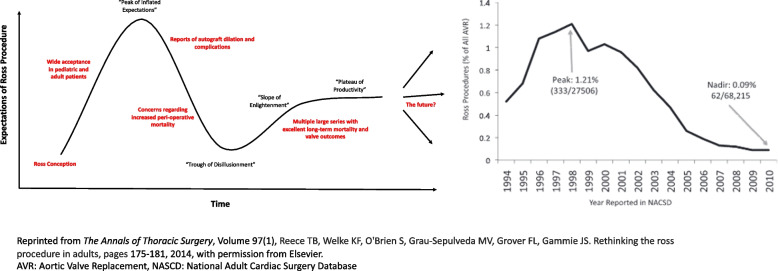
Ross utilization vs. expectations: the hype cycle

The next two domains include both the inner and outer setting, which are representative of the interactions within and between social, political, economic, structural, and cultural contexts. We inferred that these respective interactions can be observed utilizing a graph adapted from the Gartner hype cycle, which serves to represent the maturation and adoption of technologies and applications (Fig. 1) [35]. We observe that after Ross conception there was widespread adoption and enthusiasm that later peaked perhaps due to “inflated expectations” (late 1990s) before leading to a “trough of disillusionment” (2010s) after the publications on complications and post-operative mortality. This general graphical trend closely mirrors the “Ross utilization” figure from Reece et al., which is likely illustrative of the social/organizational contexts from Ross’ D&I (Fig. 1, reprinted with permission from Elsevier). Contemporary data support that Ross has improved hemodynamics, potential for excellent long-term durability, and to date has been the only operation that has resulted in restored life expectancy in young and middle-aged adults [3]. Given this, we feel that the trend of data is suggestive of a “slope of enlightenment”, where accrual of further technical and center experience has led to an improved understanding of Ross’ optimal performance and utilization. It remains unclear what the future holds, and it is imperative that the cardiovascular community not repeat some of the patterns from decades past that led to poor outcomes. In general, effective innovations that receive widespread adoption and acceptance enter a prolonged phase of the “plateau of productivity”. The next decade of results on contemporary Ross outcomes will likely determine which direction the curve ultimately travels.

The last domains include the individuals involved with the intervention and/or implementation process, and the implementation process itself. CFIR notes that individuals each possess agency and can influence others, notably through organizations and professional mindsets, norms, and affiliations, and that active change is achieved when individuals and organizations use the intervention as designed [9]. Inherent to any new procedure or technology is a learning curve, where adaptation and refinement leads to greater fidelity of an interventions intended use. We observe that while there is some cultural and organizational shift as evidenced by slow incorporation into guidelines, a notable gap persists between the history and D&I of the Ross procedure and its future. There are likely several explanations for continued resistance towards acceptance of the Ross procedure within this context. First, there is a relatively technically straightforward alternative to the Ross procedure in adult patients, notably conventional AVR. As mentioned, the Ross is a technically demanding operation and many potential pitfalls exist. Further, the Ross includes a root replacement which involves the reimplantation of coronary artery buttons. This is unique compared to AVR. Additionally by harvesting a pulmonary autograft and reconstructing the RVOT, this introduces the potential for needing re-interventions on two valves in the future. Finally, the introduction of transcatheter aortic valve replacement (TAVR) technology has led to increasing enthusiasm towards using bioprosthetic valves in a younger patient population with future hopes of valve-in-valve (ViV) TAVR. We believe much of this change in paradigm is premature, as there remains little data regarding long-term outcomes regarding valve-in-valve TAVR in young patients. Future studies will look to elucidate whether such an approach is capable of alleviating the excess mortality observed in young patients post-conventional AVR.

## Discussion

### What is the missing bridge?

Several components have been recognized within the past decade as critically important to the success of an adult Ross program. The first is appropriate surgical expertise that includes a broad aortic valve/root skill set and/or adult congenital experience. In many circumstances, this can be achieved by forming partnerships and collaborations with local adult and congenital heart surgeons. This is accompanied by performing a requisite number of Ross procedures, which typically requires seeing a significant number of younger bicuspid aortic valve (BAV) aortic valve patients and further highlights the importance of the center volume-outcome relationship. The “learning curve” associated with the Ross procedure has been observed to be around 75-100 cases, and thus very low-volume programs will likely never achieve this threshold in a reasonable timeframe [36]. Technical modifications of the Ross procedure have also likely improved recent outcomes. These include aortic annular/sinotubular junction stabilization (with the use of dacron grafts and “rings”), use of a “protected” Ross in “high-risk” settings (dilated aortic annulus, primary aortic regurgitation indication, etc.), trimming of the autograft to minimize pulmonary artery length, deep implantation of the autograft within the left ventricular outflow tract, and use of decellularized pulmonary homografts. Finally, perhaps the most impactful intervention was the relationship between post-operative hypertension and subsequent autograft dilation. This has led to very aggressive blood pressure monitoring up to a year out from surgery. Through the prevention of hypertension (systolic blood pressure > 110mmHg), it has been demonstrated that early autograft dilation can be substantially slowed or even prevented [37].

Effective surgeon training and collaboration are extremely important. It is imperative that leaders within this space are open to sharing their knowledge and expertise so that others in the earlier phases of program development might avoid the issues seen in the past. To this end, the prospective data monitoring and audit process is essential. A widespread “Ross database” with obligate outcome reporting for Ross centers of excellence (COE) would help to establish accurate outcomes data on a widespread scale to help truly understand “effectiveness” (as opposed to purely efficacy) in the real world.

**Fig. 2:**
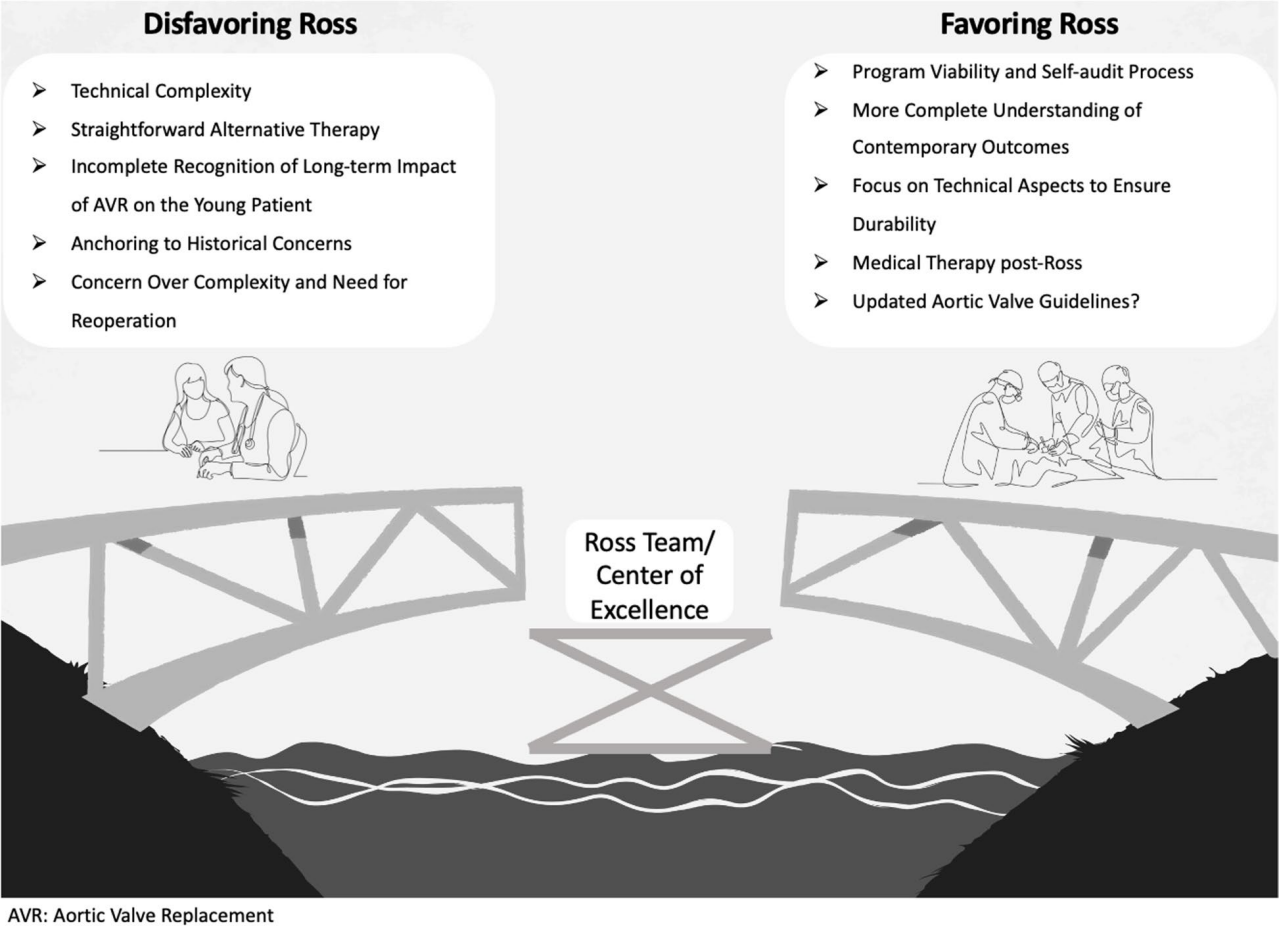
The Ross troubled waters: what’s the missing bridge

Finally, the importance of an entire Ross team concept cannot be overstated. Unique peri-operative management is required post-Ross and appropriate institutional expertise is required. Stringent blood pressure control and autograft monitoring with serial imaging require significant coordination and infrastructure, and a team approach is critical to engage multiple stakeholders in this process. It is through this engagement that active change can be achieved and a Ross program can be successfully implemented with its intended fidelity. We believe it is through this combination of multi-faceted and multi-contextual factors that the “missing bridge” between reputational concerns regarding Ross (likely reflected in current guidelines) and excellent long-term outcomes that has recently been published from centers worldwide (Fig. 2).

In summary, with proper patient selection and center experience, the Ross procedure likely has long-term benefits in non-elderly patients with valvular heart disease and may in fact restore patients to a “normal” life expectancy. The historical D&I of the Ross procedure was a result of inadequate appreciation of the subtly of technical complexity and effect of patient selection and complex aortic surgery experience, resulting in the dismissal of an efficacious procedure due to a misunderstanding of effectiveness. Contemporary data repeatedly show the long-term excellent outcome of the Ross procedure in young and middle-aged adults. Further, these same data continue to illuminate the “cost” placed on patients with conventional aortic valve replacement, which includes valve deterioration, anticoagulation/thrombotic-related event, and increased mortality. Only time will tell if aortic valve guidelines will be updated to reflect this contemporary practice. This would undoubtebly assist in mending the conflict between historical perceptions and the true effectiveness of this innovative procedure. This is the path that may ultimately help lead to a “plateau of production” as it relates to the care of aortic valvular disease in our younger patient population.

## Conclusions

The D&I of the Ross procedure was a result of an inadequate appreciation of technical complexity, effect of patient selection, and complex aortic surgery experience, resulting in the dismissal of an efficacious procedure due to a misunderstanding of effectiveness.

## Data Availability

Not applicable.

## Abbreviations

D&I: Dissemination and implementation
mAVR: Mechanical aortic valves
CFIR: Consolidated Framework for Implementation Research
AVR: Aortic valve replacement
AHA/ACC: American Heart Association/American College of Cardiology
ESC: European Society of Cardiology
RVOT: Right ventricular outflow tract
TAVR: Transcatheter aortic valve replacement
ViV: Valve-in-valve
BAV: Bicuspid aortic valve
COE: Centers of excellence

## References

[1] Eccles MP, Mittman BS (2006). Welcome to Implementation Science. Implementation Sci.

[2] Glasgow RE, Vinson C, Chambers D, Khoury MJ, Kaplan RM, Hunter C (2012). National Institutes of Health approaches to dissemination and implementation science: current and future directions. Am J Public Health.

[3] Heiden BT, Tetteh E, Robbins KJ (2022). Dissemination and Implementation Science in Cardiothoracic Surgery: A Review and Case Study. Ann Thorac Surg.

[4] Proctor E, Silmere H, Raghavan R (2011). Outcomes for implementation research: conceptual distinctions, measurement challenges, and research agenda. Adm Policy Ment Health.

[5] Proctor EK, Landsverk J, Aarons G, Chambers D, Glisson C, Mittman B (2009). Implementation research in mental health services: an emerging science with conceptual, methodological, and training challenges. Adm Policy Ment Health.

[6] Mazine A, Rocha RV, El-Hamamsy I (2018). Ross Procedure vs Mechanical Aortic Valve Replacement in Adults: A Systematic Review and Meta-analysis. JAMA Cardiol.

[7] Reece TB, Welke KF, O'Brien S, Grau-Sepulveda MV, Grover FL, Gammie JS (2014). Rethinking the ross procedure in adults. Ann Thorac Surg.

[8] Klieverik LM, Takkenberg JJ, Bekkers JA, Roos-Hesselink JW, Witsenburg M, Bogers AJ (2007). The Ross operation: a Trojan horse?. Eur Heart J.

[9] Damschroder LJ, Aron DC, Keith RE, Kirsh SR, Alexander JA, Lowery JC (2009). Fostering implementation of health services research findings into practice: a consolidated framework for advancing implementation science. Implement Sci.

[10] Ross DN (1967). Replacement of aortic and mitral valves with a pulmonary autograft. Lancet.

[11] Rabkin-Aikawa E, Aikawa M, Farber M (2004). Clinical pulmonary autograft valves: pathologic evidence of adaptive remodeling in the aortic site. J Thorac Cardiovasc Surg.

[12] Stelzer P, Jones DJ, Elkins RC (1989). Aortic root replacement with pulmonary autograft. Circulation..

[13] David TE, Omran A, Webb G, Rakowski H, Armstrong S, Sun Z (1996). Geometric mismatch of the aortic and pulmonary roots causes aortic insufficiency after the Ross procedure. J Thorac Cardiovasc Surg.

[14] Mokhles MM, Rizopoulos D, Andrinopoulou ER (2012). Autograft and pulmonary allograft performance in the second post-operative decade after the Ross procedure: insights from the Rotterdam Prospective Cohort Study. Eur Heart J.

[15] Takkenberg JJ, Klieverik LM, Schoof PH (2009). The Ross procedure: a systematic review and meta-analysis. Circulation.

[16] Hokken RB, Takkenberg JJ, van Herwerden LA, Roelandt JR, Bogers AJ (2003). Excessive pulmonary autograft dilatation causes important aortic regurgitation. Heart.

[17] Mazine A, El-Hamamsy I (2020). Procedures and Outcomes of Surgical Aortic Valve Replacement in Adults. Cardiol Clin.

[18] Hughes GC, Zhao Y, Rankin JS (2013). Effects of institutional volumes on operative outcomes for aortic root replacement in North America. J Thorac Cardiovasc Surg.

[19] Nishimura RA, Otto CM, Bonow RO (2017). 2017 AHA/ACC Focused Update of the 2014 AHA/ACC Guideline for the Management of Patients With Valvular Heart Disease: A Report of the American College of Cardiology/American Heart Association Task Force on Clinical Practice Guidelines. J Am Coll Cardiol.

[20] Baumgartner H, Falk V, Bax JJ (2017). 2017 ESC/EACTS Guidelines for the management of valvular heart disease. Eur Heart J.

[21] Hammermeister K, Sethi GK, Henderson WG, Grover FL, Oprian C, Rahimtoola SH (2000). Outcomes 15 years after valve replacement with a mechanical versus a bioprosthetic valve: final report of the Veterans Affairs randomized trial. J Am Coll Cardiol.

[22] Stassano P, Di Tommaso L, Monaco M (2009). Aortic valve replacement: a prospective randomized evaluation of mechanical versus biological valves in patients ages 55 to 70 years. J Am Coll Cardiol.

[23] Oxenham H, Bloomfield P, Wheatley DJ (2003). Twenty year comparison of a Bjork-Shiley mechanical heart valve with porcine bioprostheses. Heart.

[24] Appoo JJ, Bozinovski J, Chu MW (2016). Canadian Cardiovascular Society/Canadian Society of Cardiac Surgeons/Canadian Society for Vascular Surgery Joint Position Statement on Open and Endovascular Surgery for Thoracic Aortic Disease. Can J Cardiol.

[25] Goldstone AB, Chiu P, Baiocchi M (2017). Mechanical or Biologic Prostheses for Aortic-Valve and Mitral-Valve Replacement. N Engl J Med.

[26] Mazine A, David TE, Rao V (2016). Long-Term Outcomes of the Ross Procedure Versus Mechanical Aortic Valve Replacement: Propensity-Matched Cohort Study. Circulation.

[27] Buratto E, Shi WY, Wynne R (2018). Improved Survival After the Ross Procedure Compared With Mechanical Aortic Valve Replacement. J Am Coll Cardiol.

[28] Gofus J, Fila P, Drabkova S (2022). Ross procedure provides survival benefit over mechanical valve in adults: a propensity-matched nationwide analysis. Eur J Cardiothorac Surg.

[29] El-Hamamsy I, Toyoda N, Itagaki S (2022). Propensity-Matched Comparison of the Ross Procedure and Prosthetic Aortic Valve Replacement in Adults. J Am Coll Cardiol.

[30] Mastrobuoni S, de Kerchove L, Solari S (2016). The Ross procedure in young adults: over 20 years of experience in our Institution. Eur J Cardiothorac Surg.

[31] Sievers HH, Stierle U, Charitos EI (2016). A multicentre evaluation of the autograft procedure for young patients undergoing aortic valve replacement: update on the German Ross Registry. Eur J Cardiothorac Surg.

[32] Sievers HH, Stierle U, Petersen M (2018). Valve performance classification in 630 subcoronary Ross patients over 22 years. J Thorac Cardiovasc Surg.

[33] David TE, Ouzounian M, David CM, Lafreniere-Roula M, Manlhiot C (2019). Late results of the Ross procedure. J Thorac Cardiovasc Surg.

[34] Pergola V, Di Salvo G, Fadel B (2020). The long term results of the Ross procedure: The importance of candidate selection. Int J Cardiol.

[35] Gartner Hype Cycle. Interpreting technology hype. https://www.gartner.com/en/research/methodologies/gartner-hype-cycle. Accessed 8 Nov 2022.

[36] Bouhout I, Ghoneim A, Poirier N (2017). Impact of the Learning Curve on Early Outcomes Following the Ross Procedure. Can J Cardiol.

[37] Mazine A, El-Hamamsy I, Verma S (2018). Ross Procedure in Adults for Cardiologists and Cardiac Surgeons: JACC State-of-the-Art Review. J Am Coll Cardiol.

